# Examining the Role of Food Form on Children's Self-Regulation of Energy Intake

**DOI:** 10.3389/fnut.2022.791718

**Published:** 2022-02-09

**Authors:** Nicole A. Reigh, Barbara J. Rolls, Lori A. Francis, Kristin A. Buss, John E. Hayes, Marion M. Hetherington, Kameron J. Moding, Samantha M. R. Kling, Kathleen L. Keller

**Affiliations:** ^1^The Metabolic Kitchen and Children's Eating Behavior Laboratory, Department of Nutritional Sciences, The Pennsylvania State University, University Park, PA, United States; ^2^The Laboratory for the Study of Human Ingestive Behavior, Department of Nutritional Sciences, The Pennsylvania State University, University Park, PA, United States; ^3^Center for Family Research in Diverse Contexts, Department of Biobehavioral Health, The Pennsylvania State University, University Park, PA, United States; ^4^The Emotion Development Laboratory, Departments of Psychology and Human Development and Family Studies, The Pennsylvania State University, University Park, PA, United States; ^5^Department of Food Science, Sensory Evaluation Center, The Pennsylvania State University, University Park, PA, United States; ^6^Human Appetite Research Unit, School of Psychology, Woodhouse, The University of Leeds, Leeds, United Kingdom; ^7^Child Temperament and Health Laboratory, Department of Human Development and Family Studies, Purdue University, West Lafayette, IN, United States; ^8^Evaluation Sciences Unit, Division of Primary Care Population Health, Department of Medicine, School of Medicine, Stanford University, Stanford, CA, United States; ^9^The Metabolic Kitchen and Children's Eating Behavior Laboratory, Departments of Nutritional Sciences and Food Science, The Pennsylvania State University, University Park, PA, United States

**Keywords:** pediatric obesity, energy compensation, preload, food form, self-regulation

## Abstract

Increasing childhood obesity rates in both the United States and worldwide demonstrate a need for better prevention and intervention strategies. However, little is understood about what factors influence children's ability to sense and respond to hunger and fullness cues, a critical component of self-regulation of energy intake and maintenance of a healthy body weight. Research in adults suggests that food form may influence self-regulation of energy intake. More specifically, beverages are not as satiating as solid foods when matched for factors such as energy content, energy density, and volume and therefore elicit poorer energy intake self-regulation. However, much less is known about the impact of food form on children's ability to regulate their energy intake. This report describes a study that will examine the relationship between biological, cognitive, and psychological factors and children's appetite self-regulation (ASR). In this registered report, we will examine the influence of food form on children's short-term energy compensation, a proxy indicator of energy intake self-regulation. The study will employ a within-subjects, crossover design in which children (*n* = 78) ages 4.5–6 years will attend five laboratory visits, each ~1 week apart. During each visit, children will be presented with one of five possible preload conditions: apple slices, apple sauce, apple juice, apple juice sweetened with non-nutritive sweetener (NNS), or no preload. The order of preload conditions will be pseudorandomized and counterbalanced across participants. Following consumption of the preload (or no preload), children will consume a standardized *ad libitum* test meal of common foods for this age group. We hypothesize that children will demonstrate poorer short-term energy compensation (greater meal intake) in response to the liquid and semi-solid preloads compared to the solid preload. Understanding how energy in various forms affects children's ability to self-regulate intake has implications for dietary recommendations and will help identify those who are most at-risk for poor intake regulation and the development of obesity.

## Introduction

As childhood obesity rates continue to increase in both the United States (1) and worldwide (2) it is imperative to understand why some children eat beyond their energy needs, as this has been identified as a behavioral phenotype for obesity (3). One factor that may contribute to a positive energy balance is poor appetite self-regulation (ASR) (4), conceptualized in recent reviews as a multi-faceted construct characterizing the ability to regulate energy intake in response to biological, social, and psychological influences (5). Early studies that primarily assessed homeostatic influences on energy regulation found that infants can upregulate their energy intake in response to energy deficits (6, 7). However, this ability declines with age (8) and by the time children reach preschool (i.e., 3–5 years), they are less able to regulate energy intake in response to environmental perturbations (e.g., portion size, energy density, parenting practices) (9–13). These more recent studies reinforced the notion that ASR is influenced by more than just homeostatic signals coming from the gut and periphery. The current registered report describes the methods for a study intended to examine the interplay between social, psychological, and biological factors on children's ASR.

**Figure 1:**
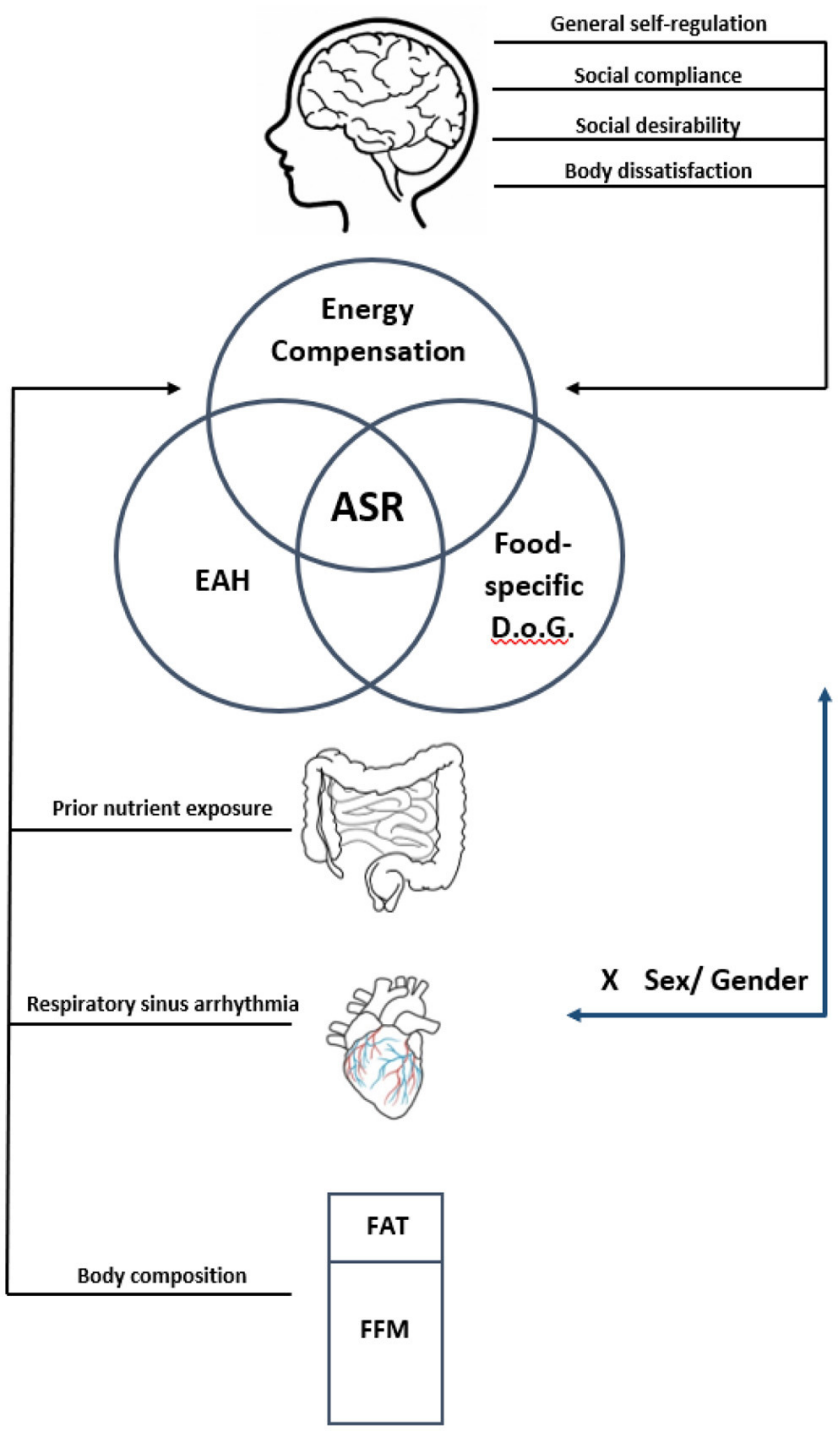
Dual-process model of psychosocial and physiological factors hypothesized to influence ASR. This study posits that ASR involves both bottom-up and top-down processes that may be influenced by a variety of factors. ASR itself is to be operationalized using common paradigms to assess energy compensation, eating in the absence of hunger (EAH) and food-specific delay of gratification (D.o.G). The factors hypothesized to influence bottom-up processes include previous exposure to non-nutritive sweeteners, respiratory sinus arrythmia, and body composition, specifically the ratio of fat mass to fat-free mass (FFM). Top-down influences to be tested include measures of general self-regulation including social compliance and social desirability, as well as body dissatisfaction. We also hypothesize that child sex, which may play a role in ASR, may also be associated with differences in both top-down and bottom-up influences on ASR.

In developing this study, we drew inspiration from reviews by Russell and Russell (4, 14) to develop a dual-process model of ASR in children (see Figure 1). In this model, ASR is influenced by bottom-up signals from the gut and periphery that send information about hunger and satiety and by top-down processes that enable children to appropriately respond to these signals by controlling the amount of energy consumed. It is likely that many factors, including child-level individual differences (e.g., sex/gender, appetitive traits, general self-regulation, social desirability) and food characteristics (i.e., portion size, food form) can either enhance or disrupt children's ASR. In order to develop a more comprehensive picture of children's ASR, we operationalized it as a combination of (1) energy compensation (i.e., the ability to regulate energy intake in response to food form and energy content), (2) eating in the absence of hunger (EAH), and (3) food-specific delay of gratification. Energy compensation was selected as the primary outcome because it is the gold-standard for measuring satiety in response to manipulations in food form or energy content, and it captures the interplay between top-down and bottom-up processes. EAH and food-specific delay of gratification were added as secondary outcomes to allow for characterization of a more complete ASR phenotype. EAH has been characterized as a measure of bottom-up approach tendencies toward food, while delay of gratification depicts top-down control over food intake (4). While outcomes related to the broader construct of ASR will be published in other reports, the current registered report will focus only on outcomes related to children's ability to regulate in response to food form and energy content, referred to as “energy compensation” ability.

The energy compensation paradigm is thought to measure children's ability to eat in response to satiety signals (14). In this paradigm, short-term energy compensation is measured by providing children with a preload that varies by some attribute, often energy density, and assessing subsequent intake at an *ad libitum* meal following a predetermined interval (15). Using this procedure, energy compensation can be quantified by comparing energy intake following the various preload conditions. Most often, preloads vary in energy content by using a non-nutritive sweetener (NNS) in the low-energy preload to match the taste, volume, and orosensory attributes of the high-energy preload. The energy content of the preloads is then masked from participants to determine how well they sense and respond to the energy content by regulating their subsequent intake at a meal. Those with “good” compensation adjust their subsequent meal intake commensurate with the energy intake from the preloads. The appeal of this approach is that the ability to regulate energy intake can be quantified in an objective manner.

Most common in the children's literature, energy compensation has been depicted as a linear transformation of the difference in intake across two preload conditions that vary by energy content (i.e., compensation index; COMPx) (3, 16–19). Studies using this approach have found that COMPx varies widely between children and differs by certain characteristics such as satiety responsiveness (20), food responsiveness (21), BMI z-score (9), age (8), and sex (16, 19, 22, 23). However, these findings are not consistently observed across studies, and relatively little is understood about the influence of other physiological, cognitive, and environmental factors on children's energy compensation, and ASR more generally.

Research conducted in adults has found a consistent effect of food form on satiety such that beverages, when matched for key factors such as weight and energy content, produce weaker satiety than solid foods (24, 25). Notably, Flood-Obbagy and Rolls (26) conducted a preloading study in which adults consumed apples in various forms (apple slices, apple sauce, and apple juice) or no preload prior to a standardized *ad libitum* meal. They found that apple slices produced greater satiety and reduced subsequent meal intake relative to both apple sauce and juice. This was despite preloads being matched for weight, fiber, energy content, energy density, and ingestion rate (26). These results align with other preloading studies that find poorer energy compensation following consumption of beverages compared to solid foods (24, 25). Additionally, one RCT in adults found that healthy-weight participants gained weight over an 8-week period when fruits and vegetables were given as a liquid compared to an 8-week period when these foods were given as solids (27). This suggests that food form may influence ASR and subsequent weight gain over longer periods of time than is typically measured in a laboratory. Several physiological mechanisms have been proposed that may help explain these findings. Relative to liquids, solid foods increase gastric distension (28) and decrease gastric emptying rate (29–31), both of which may increase satiety. Additionally, solid foods require greater mastication and oral exposure time than liquids, which may increase satiation (32, 33). Though more research is needed to understand the underlying mechanisms, the effect of food form on satiation and satiety has been consistently demonstrated in adults.

Whereas a substantial body of research in adults suggests that solid foods provide greater satiety than beverages, little is known about when these differences develop. To date, only one study has examined the impact of food form on satiety in children. Schwartz and colleagues (34) compared apple slices to apple sauce (matched for energy and energy density) and found no effect of food form on subsequent food intake in 8- to 10-year-old children (34). This study, however, did not include a liquid (beverage) preload, which is a limitation, as much of the adult literature examining the effect of food form employs the use of beverage preloads as a comparator to solid preloads. Caloric beverages (e.g., fruit juices, sports drinks) are ubiquitous in children's diets (35) and contribute ~175 kcal/day to total energy intake (36). Despite this, the effect of beverages on children's satiety relative to solid and semi-solid foods is understudied. Additionally, neither Schwartz et al. (34) nor existing adult literature has controlled for the effect of perceived volume on satiety. Solid foods appear greater in volume and thus are expected to be more satiating than beverages (37, 38), and adults have demonstrated that expected satiety before a meal may influence self-reported fullness after the meal (38). By masking volume, this study reduces the potential cognitive influences in order to better isolate the sensory and physiological effects of food form on satiety.

This study aims to address these gaps by examining the effect of food form on children's short-term energy compensation. In an effort to capture the developmental window where children are becoming less responsive to internal cues (39–41) and more responsive to external cues (9–13), we are conducting this study in 4.5- to 6-year-old children. Additionally, although oral development such as mastication efficiency shows a general increase across childhood, it appears to plateau between the ages of 4–6 years (42). This suggests that studying the effect of food form in this age group will be less confounded by age differences in oral development. Apples will be presented to children in various forms (i.e., apple slices, apple juice, and apple sauce) prior to a standardized *ad libitum* test meal. Apple preloads will be matched for weight, energy content, energy density, and total consumption time, and volume will be disguised. In addition to the apple preloads, a low-calorie apple juice sweetened with non-nutritive sweetener (NNS apple juice) and a no preload condition will be included. The first aim of this study is to examine the effect of apples in various forms on children's subsequent energy intake at a standardized test meal. Specifically, we hypothesize that the regular apple juice will elicit the poorest energy compensation relative to both apple sauce (semi-solid) and apple slices (solid). Additionally, we hypothesize that apple sauce will result in greater satiety than apple juice. The second objective of this study is to examine energy compensation within the same food form by comparing meal intakes following the regular apple juice and NNS apple juice. We hypothesize that children will consume less following the regularly sweetened apple juice than the apple juice sweetened with NNS.

## Methods

### Experimental Design

**Figure 2:**
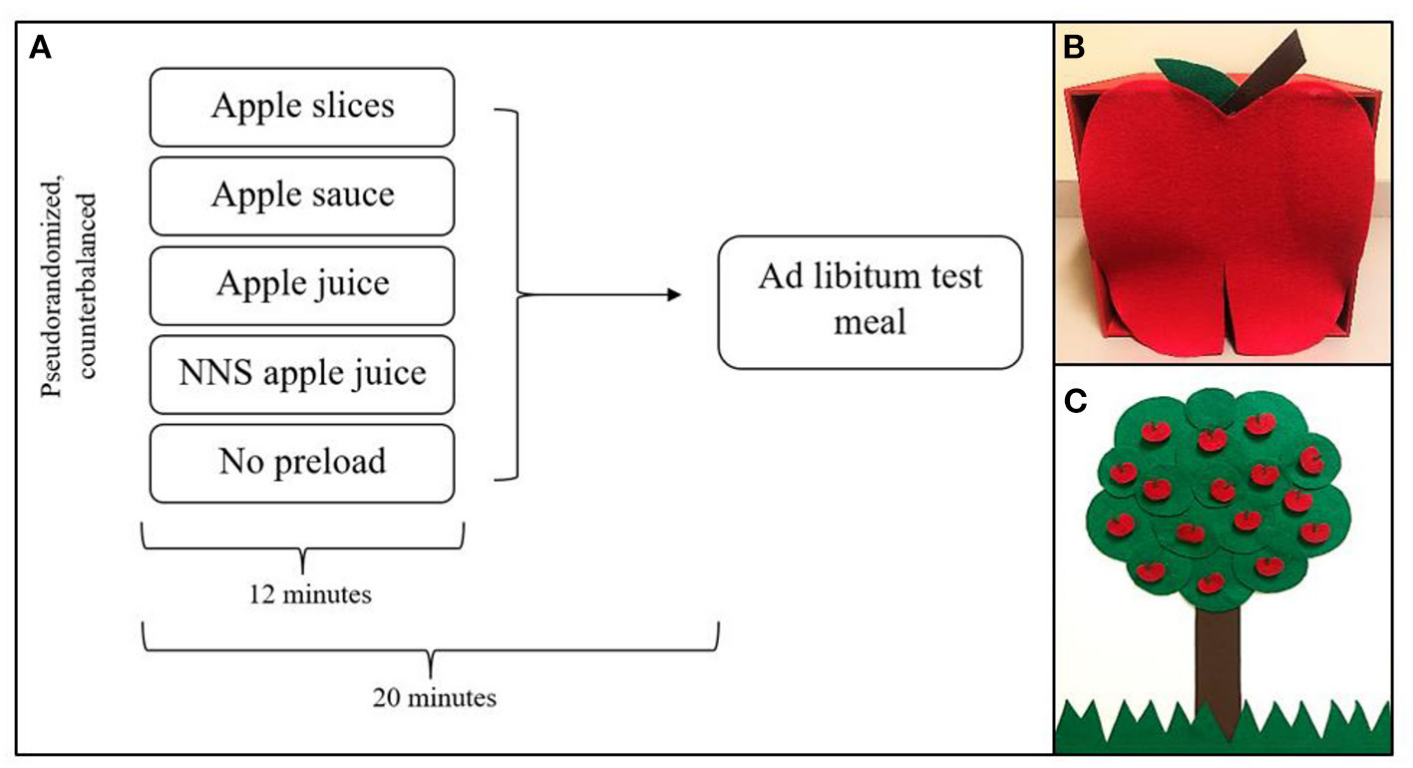
Overview of preload protocol **(A)**, child-friendly box to mask preload volume **(B)**, and game board to encourage children to finish preloads **(C)**.

This study will use a within-subjects, crossover design with repeated measures. Children will visit the laboratory once a week over an approximate 5-week period, for a total of five, one and a half hour sessions. Families will attend either lunch or dinner meals based on availability, but meal times will be kept consistent within families. Children will be fasted for 3 h upon arrival to each visit. On each test session, children will be served one of five preload conditions: apple slices, apple sauce, regular apple juice, NNS apple juice, or no preload. The same standardized test meal will then be served ~20 min after the start of preload consumption, a timeframe chosen based on previous research in preschool-aged children (16, 43) and to account for the rapid pace in which liquids are emptied from the stomach (44). On the final visit, children's EAH will be assessed following their test meal. Therefore, the order in which preloads will be delivered to children will be pre-established from a limited number of possible orders so that one of the three caloric preloads is delivered prior to children's EAH assessment. These orders will be counterbalanced across participants. Figure 2A provides an overview of the preloading protocol. This study was approved by the Institutional Review Board of The Pennsylvania State University (IRB #13957) in accordance with the Declaration of Helsinki. On visit 1, parents give informed consent to allow their children to participate in the study.

### Participants

Inclusion criteria for the study will be children between the ages of 4.5–6 years-old who are physically healthy, with no food allergies or medical conditions that affect appetite or ability to follow study protocol. Excluded medical conditions include children with autism or developmental delays, many of whom are prone to feeding difficulties (45). Children must also like and be willing to consume apple slices, apple sauce, and apple juice in addition to at least four of the five *ad libitum* test meal foods, according to parental report on a screening questionnaire. The legal parent or guardian primarily responsible for child feeding decisions must be able to attend all visits with the child. While no recruitment restrictions will be based on children's weight status or race/ethnicity, we expect the majority of children to be white, non-Hispanic or Latinx, and of healthy weight status (BMI-for-age percentile <85) based on recent census data from where the study will be conducted (46). Based on the same census data, we expect children to come from relatively affluent families, as median household income for families with children ranges from ~$93,00–107,000 per year (46). As child sex may play an important role in ASR (12, 16, 19, 22, 23, 47), and our study aims to further examine these sex differences, even numbers of boys and girls will be recruited.

### Energy Compensation

Energy compensation will be examined by comparing meal intake following each preload condition. According to the pre-established order, children will consume one of four preloads or no preload (five possible conditions) at the beginning of each visit. A no preload condition will be included to assess children's usual intake without a pre-meal snack; the four remaining preloads will be apple slices, applesauce, regular apple juice, and apple juice sweetened with NNS. Apple slices, applesauce, and regular apple juice will be matched for energy content (63.8 kcal), weight (133.0 g), and energy density (0.48 kcal/g). The NNS preload will be matched for weight with the other preloads but, by design, will not be matched for energy content or energy density (Table 1).

**Table 1:** Weight, energy content, energy density, and macronutrient composition of preloads and standardized *ad libitum* test meal foods.

	**Weight** **(g)**	**Energy** **(kcal)**	**Energy density** **(kcal/g)**	**Carbohydrate** **(g)**	**Fiber** **(g)**	**Fat** **(g)**	**Protein** **(g)**
**Preloads**							
Apple slices, peeled	133.0	63.8	0.48	15.3	1.7	0.0	0.0
Applesauce with peeled apples	133.0	63.8	0.48	15.3	1.7	0.0	0.0
Regular Apple juice	133.0	63.8	0.48	14.9	0.5	0.0	0.0
NNS Apple juice	133.0	16.5	0.12	3.8	0.0	0.0	0.0
No preload	–	–	–	–	–	–	–
***Ad libitum*** **test meal (first serving)**							
Macaroni and cheese	100.0	198.0	1.98	27.2	1.1	6.2	5.7
Broccoli	61.0	26.5	0.43	1.8	1.8	0.0	0.9
Baby carrots	35.0	12.3	0.35	1.9	1.0	0.0	0.0
Grapes	113.0	75.7	0.67	18.4	1.0	0.0	0.7
Graham crackers	~23.0	97.5	4.24	17.3	0.8	2.6	1.5
Water	226.8	0.0	0.00	0.0	0.0	0.0	0.0

Apples were chosen as they are widely accepted by children and are commonly consumed in various forms (48). In addition, this choice will allow comparison with prior studies done in adults (26) and children (34). Preload amounts were selected based on the average weight of apples consumed per eating occasion in a similar age group using a nationally-representative sample (49). Apple sauce and apple juice recipes were developed based on Flood-Obbagy and Rolls' study in adults (26). Apple slices (133 g) will be served to children without the skin in order to increase acceptance and avoid additional fiber that could impact satiety. Apple sauce will be prepared using 133 g peeled, sliced apples heated for 20 min at 350 degrees Fahrenheit. Once cooked, the apples will be puréed and any water lost during heating will be added back to ensure consistent weight between preload conditions. Apple juice (133 g) will be 120.4 g of Mott's 100% Apple Juice (Mott's® Mott's Inc., Plano, TX) and 12.6 g water to match the weight and energy density of the apple slices and apple sauce. Lastly, NNS apple juice (133 g) will be prepared with 66.5 g Mott's Light Apple Juice (Mott's® Mott's Inc., Plano, TX) and 66.5 g Old Orchard Healthy Balance Diet Apple Juice Cocktail (Old Orchard Brands LLC® Lassonde Industries Inc., Sparta, MI). This was done because informal taste testing among 14 research assistants revealed that the diet juice cocktail alone was not palatable; however, a mixture of the diet juice cocktail and regularly sweetened apple juice improved palatability. In order to create a product that was both palatable and low in energy compared to the regular apple juice, the diet juice cocktail was combined with the “light” version of the same brand of apple juice used in the regular apple juice condition.

Energy compensation by food form will be assessed by comparing meal intake following each of the caloric preloads (apple slices, apple sauce, regular apple juice), discussed in more detail in the “Data Analysis” section. Additionally, in order to better compare these findings to the broader literature, COMPx (3, 16–19) will be calculated using data from the regular and NNS apple juice conditions. The equation to calculate COMPx is:


$$\begin{array}{l} \frac{\text{Meal kcal following NNS juice }-\text{ Meal kcal following regular juice }}{\text{ Regular juice kcal }-\text{ NNS juice kcal }}\times 100 \end{array}$$

Using this equation, a COMPx of 100% indicates perfect compensation (adjustment) for the energy in each preload, meaning children reduced their meal intake commensurate with the energy in each preload. A COMPx above 100% indicates that children overcompensated (underate) at the meal following the regular apple juice compared to the NNS apple juice, and COMPx below 100% indicates that children undercompensated (overate) at the meal following the regular apple juice compared to the NNS apple juice.

### Preload Administration

In addition to matching the preloads for attributes of weight and energy content, we also aim to control perceived volume and ingestion time of each preload, both of which can impact satiety. Without standardizing ingestion time, the beverages would likely be consumed more quickly than the other preloads, and this would impact the interval between the preload and subsequent meal. As the time interval is an important driver of energy compensation (24) we would not be able to disentangle effects of food form from that of eating rate. Similarly, because volume or amount served can influence expected satiety, we developed methods to disguise the volume of the preloads. By doing this, we hope to reduce the impact of visual volume cues on subsequent satiety. As a result of these design choices, we expect the see less robust, but still significant, influences of food form on satiety than what have been reported in other studies (24). This outcome will help to isolate potential mechanisms whereby food form influences satiety that can be targeted in future studies.

To address differences in perceived volume across the various conditions, preloads will be disguised under a colorful, child-friendly, apple-themed box (Figure 2B). This box reduces the amount of time children spend looking at the preload and prevents them from seeing the entire volume to be consumed at once. To standardize ingestion time, an audio recording of a story will be played for the children during preload consumption. These stories were developed to mention a key word (“apple”) once every 45 s which serves as a cue for children to reach into the box and pull out a soufflé cup containing one pre-portioned amount of the preload. Each story mentions the key word 16 times, for a total of 16 equal-weight preload portions across 12 min. To encourage children to finish each preload, a research assistant will show the child an apple tree-themed game board (Figure 2C) while the audiobook plays. Each time the child consumes one of the preload portions, the research assistant will remove an apple from the tree on the game board and place it into a small basket. Children will be instructed that they must collect all of the apples from the tree in order to earn a sticker. During the no preload condition, children will listen to an audiobook that also mentions the key word “apple” 16 times, once every 45 s. However, the storyline follows a boy who is missing apples, so the children will be instructed to reach into the box to see if any apples are there. During this condition, children will also have a chance to earn stickers by collecting all of the apples from the apple tree game board. This will mimic the timing and protocol of the other preload conditions to control for these factors.

### *Ad libitum* Test Meal

Approximately 20 min after the start of preload ingestion, a standard *ad libitum* test meal will be served consisting of the following familiar, commercially-available foods: macaroni and cheese (Kraft®, Kraft Heinz Co., Chicago, IL), frozen broccoli florets (Birds Eye®, Conagra Brands, Chicago, IL), red grapes (Wegmans®, Wegmans Food Markets, Rochester, NY), baby carrots (Wegmans®, Wegmans Food Markets, Rochester, NY), graham crackers (Nabisco Original®, Nabisco, East Hanover, NJ), and water. Amount (in grams) and energy content of each test meal food are displayed in Table 1. Children will be given 30 min to eat until comfortably full and may request additional portions of any of the five test foods, if desired. Each additional portion will weigh the same as the first portion (see Table 1). Weights will be taken before and after the child's meal in order to determine total consumption of each food in grams.

### Liking and Hunger Ratings

In order to capture other factors that may influence children's intake, including how much they like the foods and variable hunger levels, we will assess liking of each preload and test meal food using a five-point hedonic scale (50). Perceived hunger ratings will be collected before and after each preload, and before and after each test meal using a four-point silhouette scale depicting varying degrees of stomach fullness (51).

### Other Measures

As this study posits that successful ASR requires both bottom-up and top-down regulatory processes, other measures of ASR as well as child-level characteristics that may either enhance or disrupt these processes will be tested and reported in future publications. These measures are summarized below and are modeled in Figure 1.

Eating in the Absence of Hunger (EAH)- Following their *ad libitum* test meal on visit 5, children's EAH will be assessed using a widely-accepted protocol (52). Twenty min after the end of their test meal, children will be presented with 6 palatable snack foods: potato chips (Lay's® original, PepsiCo, Harrison, NY), cookies (Chips Ahoy!® chocolate chip cookies, Nabisco, East Hanover, NJ), fruit candy (Starbursts® original chews, Mars Inc.®, McLean, VA), M&M's (Mars Inc.®, McLean, VA), corn chips (Fritos® The Original corn chips, PepsiCo, Harrison, NY), and brownies (Entenmann's® Little Bites Fudge Brownies, Bimbo Bakeries, Horsham). Children will also be presented with several toys and will be left alone for 10 min to eat and/or play with whatever they would like, but will not be told how long they have to do so. EAH is a common method used to assess children's food approach behaviors and tendency to eat when satiated, an aspect of ASR (4).

Delay of gratification (D.o.G.)- Children's food-specific delay of gratification will be assessed using a waiting task (53). During this task, a research assistant will first ask the child to choose which snack they would most like to play for from three possible choices: coated chocolate candies, animal crackers, or pretzels. The child will be given instructions to wait until the researcher enters the room in order to receive a larger portion of the snacks, or the child may eat the smaller portion of the snack before the researcher enters the room. The child will not be told, however, how long they must wait in order to earn the larger portion and they may ring a bell if they would like to end the task early. The researcher will exit the room after giving instructions, and this task will end when either (a) the child ends the task by eating the food or ringing the bell or (b) 7 min have passed. While performance on this task does not specifically measure energy intake regulation, it may be related to cognitive efforts to control what or how much is consumed. It is therefore implicated in the pre-consumption phase of ASR (14) and may also be related to eating cessation.

Child sex- A demographics questionnaire will be administered to parents to assess biological sex. Due to the age of the children, we are not asking parents to report gender, which is a sociological construct (54). However, as detailed in our model (Figure 1), we hypothesize that many of the psychological and social influences likely to impact ASR may also differ by or interact with child sex/gender. Several studies assessing children's energy compensation have found that boys demonstrate better COMPx than girls (16, 19, 22, 23). However, these findings are not consistent (12, 17, 18, 20, 41) and to our knowledge, no studies have been designed and powered *a priori* to examine these sex differences. An aim of this study, therefore, is to examine whether children's COMPx, calculated by comparing meal intake following the regular apple juice compared to the NNS apple juice, differs by child sex.

Anthropometrics and Body Composition- Children's height and weight will be measured at the beginning of their first laboratory visit after removing shoes, socks, and jackets. Weight will be measured to the nearest tenth of a kilogram using a digital body scale and height will be measured to the nearest half of a centimeter using a stadiometer. Each measurement will be taken in duplicate, and averaged values will be used for data analysis. BMI percentiles and z-scores will be calculated using the Center for Disease Control (CDC) age- and sex- specific BMI cutoffs. Body fat percentage (adiposity), lean body mass (fat-free mass; FFM), and bone mineral density will be measured using dual-energy X-ray absorptiometry (DXA) at the Clinical Research Center at Penn State. Lean body mass is a determinant of energy needs and has been hypothesized to be a key determinant of the ability to regulate appetite (55), and may therefore be an important bottom-up process involved in ASR.

Previous Exposure to NNS- Previous exposure to NNS will be assessed using the Beverage Questionnaire for Preschoolers (BEVQ-PS), a parent-reported measure of beverage intake that has been validated in this age group (35). Additionally, a version of the Artificial Sweetener (Non-nutritive Sweetener) Intake Questionnaire adapted to measure children's NNS intake will be collected from parents. Children with a greater exposure to non-nutritive sweeteners (NNS) may be at greater risk of overweight and obesity (56). A possible hypothesis for this could be that these children demonstrate poorer ASR, potentially due to an “uncoupling” of energy signaling and energy intake (56), but this has not yet been tested in children.

Respiratory sinus arrhythmia (RSA)- To better understand if ASR in children is dictated by physiological, and therefore subconscious, processes, respiratory sinus arrhythmia will be measured during the children's meals. RSA can be used to approximate vagal nerve activity, which could provide insight into the physiological responses to various food forms and potentially ASR more generally.

Social desirability- Two measures will be collected to approximate children's social desirability. First, a Do/Don't task (57) will be conducted on the first visit to assess children's compliance with the researcher. Following the test meal, the researcher will present the child with an assortment of age-appropriate, attractive toys from a basket and will dump the contents of the basket onto a table in front of the child. The child will have 5–10 min of free play, after which the research assistant will ask the child to put away the toys that were set out. The research assistant will then leave the room. The task ends when either (a) 3 min have passed or (b) the child has finished putting all the toys away. Once this “do” task is complete, children will complete the “don't” task. The research assistant will re-enter the room with a wrapped box containing a small toy. The child will be asked not to touch the gift box until the researcher returns, and the research assistant will leave the room. This task will last 3 min and, similarly to D.o.G, children will not be told the length of time that they must refrain from touching the gift box. After 3 min, the research assistant will re-enter the room and the task will end. Additionally, the Social Desirability Questionnaire for Children (58) will be collected. These measures approximate children's social desirability, which may increase as children's general self-regulation increases. ASR appears to decrease as general self-regulation increases (4); however, few studies have systematically examined both in children. Understanding more about the relationship between general self-regulation and ASR may provide insight into how to improve ASR.

Portion sorting task- A novel portion sorting task developed in our lab will be performed to assess children's ability to match pictures of foods of varying portion sizes. Children will be presented with 16 cards and asked to match the two cards with identical portion size. Children will play two rounds of this game (for a total of 32 cards) and will be timed on how long it takes to make all 8 matches each round. Following the matching task, children will be presented with a deck of 40 cards depicting various foods of different portion sizes and three baskets labeled “too much,” “just right,” and “too little.” Children will be instructed to imagine eating the food and place the picture in the appropriate basket based on how they think their bellies would feel after they finish that portion. Children will complete this task on Visit 1 prior to their meal, during a fasted state. This task may provide insight on children's abilities to discriminate visual food cues during the pre-consumption phase of ASR.

Body dissatisfaction- Three measures of children's body dissatisfaction will be collected following EAH on visit 5: The Weight Concerns Scale (59), Body Esteem Scale (60), and Body Image Scale (61). Eating in order to achieve a desired body type, rather than in response to homeostatic signals, may lead to poorer ASR, though this has not yet been tested in children.

### Data Analysis

Based on a power analysis conducted with GPower version 3.1, testing 78 children will be sufficient to achieve 80% power to detect significant differences (*P* < 0.05) between preload conditions. A small effect size (*f* = 0.2) was chosen to be conservative, and the correlation among repeated measures (*r* = 0.24) was based on previous data from our laboratory that used a preloading design in a similar age group (62). Given these specifications, the sample size needed to examine the main effect of food form (1 group, 3 measurements) was smaller than the sample size needed to examine interactions between experimental condition and sex/gender on children's COMPx following the two apple juice preloads (2 groups, 2 measurements). We therefore chose the larger sample size of 78 children and will recruit even numbers of boys (*n* = 39) and girls (*n* = 39) in order to examine sex differences.

Energy intake from the preloads and test meals will be calculated by multiplying gram intake by the energy densities outlined in Table 1. To test the hypothesis that food form affects subsequent energy intake at the test meal, a mixed linear model with repeated measures will be used to analyze the main outcomes of meal energy intake (in kilocalories) with preload type (solid, semi-solid, liquid) as the fixed factor and participant as a random factor. Additionally, mean COMPx in response to the apple juice preloads will be calculated using the aforementioned COMPx equation (43). If preload type is a significant predictor of meal energy intake, Tukey's test will be conducted to determine which condition(s) are driving these differences. To test the hypothesis that children will eat less at the meal following the regular apple juice preload compared to the NNS apple juice preload, a similar mixed linear model with repeated measures will treat preload type (high- or low-energy density) as the fixed factor and participant as a random factor. Both models will control for child sex, age, and body weight as well as preload order. Additionally, a separate model that adds pre-meal hunger as a covariate will be conducted for each hypothesis. Because differences in perceived hunger are related to our primary outcome, we will run the analyses with and without adjusting for pre-meal hunger to see if there is an independent effect of food form or energy density on subsequent intake independent of physiological hunger. Similarly, a separate model that adds food liking as a covariate will be conducted for each hypothesis as well to test the independent effect of children's liking of the test foods on energy intake. Significance will be set at α = 0.05 and all analyses will be conducted using the most recent version of the Statistical Package for the Social Sciences (SPSS Inc., Chicago, IL).

## Data Availability Statement

The original contributions presented in the study are included in the article/supplementary material, further inquiries can be directed to the corresponding author/s.

## Ethics Statement

This study was reviewed and approved by the Institutional Review Board of The Pennsylvania State University (IRB #13957) in accordance with the Declaration of Helsinki. The parents give informed consent to allow their children to participate in the study.

## Author Contributions

NR and KK were responsible for experimental design and manuscript preparation. BR, LF, KB, JH, MH, KM, and SK were responsible for experimental design and manuscript editing. All authors contributed to the article and approved the submitted version.

## Funding

This study was funded by the Pennsylvania State University Social Science Research Institute Level 2 Seed grant funding. Publication funds are provided by the Department of Nutritional Sciences of the Hershey Company endowment.

## Conflict of Interest

The authors declare that the research was conducted in the absence of any commercial or financial relationships that could be construed as a potential conflict of interest.

## Publisher's Note

All claims expressed in this article are solely those of the authors and do not necessarily represent those of their affiliated organizations, or those of the publisher, the editors and the reviewers. Any product that may be evaluated in this article, or claim that may be made by its manufacturer, is not guaranteed or endorsed by the publisher.
